# TRPV1 Antagonism by Capsazepine Modulates Innate Immune Response in Mice Infected with *Plasmodium berghei* ANKA

**DOI:** 10.1155/2014/506450

**Published:** 2014-08-24

**Authors:** Elizabeth S. Fernandes, Carolina X. L. Brito, Simone A. Teixeira, Renato Barboza, Aramys S. dos Reis, Ana Paula S. Azevedo-Santos, Marcelo Muscará, Soraia K. P. Costa, Claudio R. F. Marinho, Susan D. Brain, Marcos A. G. Grisotto

**Affiliations:** ^1^Universidade CEUMA, 65075-120 São Luís, MA, Brazil; ^2^Cardiovascular Division, King's College London, London, UK; ^3^Universidade de São Paulo, São Paulo, Brazil; ^4^Universidade Federal de São Paulo, Diadema, Brazil; ^5^Universidade Federal do Maranhão, São Luís, Brazil; ^6^Instituto Florence de Ensino Superior, São Luís, Brazil

## Abstract

Thousands of people suffer from severe malaria every year. The innate immune response plays a determinant role in host's defence to malaria. Transient receptor potential vanilloid 1 (TRPV1) modulates macrophage-mediated responses in sepsis, but its role in other pathogenic diseases has never been addressed. We investigated the effects of capsazepine, a TRPV1 antagonist, in malaria. C57BL/6 mice received 10^5^ red blood cells infected with *Plasmodium berghei* ANKA intraperitoneally. Noninfected mice were used as controls. Capsazepine or vehicle was given intraperitoneally for 6 days. Mice were culled on day 7 after infection and blood and spleen cell phenotype and activation were evaluated. Capsazepine decreased circulating but not spleen F4/80^+^Ly6G^+^ cell numbers as well as activation of both F4/80^+^and F4/80^+^Ly6G^+^ cells in infected animals. In addition, capsazepine increased circulating but not spleen GR1^+^ and natural killer (NK) population, without interfering with natural killer T (NKT) cell numbers and blood NK and NKT activation. However, capsazepine diminished CD69 expression in spleen NKT but not NK cells. Infection increased lipid peroxidation and the release of TNF*α* and IFN*γ*, although capsazepine-treated group exhibited lower levels of lipid peroxidation and TNF*α*. Capsazepine treatment did not affect parasitaemia. Overall, TRPV1 antagonism modulates the innate immune response to malaria.

## 1. Introduction

Malaria is an infectious disease caused by intracellular protozoans of the genus* Plasmodium* and transmitted from person to person through bites of infected mosquitoes. It affects millions of people annually and is a leading cause of child mortality in underdevelopment countries [1]. Severe malaria such as cerebral malaria is frequently fatal and outcome of infection depends on host's immune response, with innate immunity playing a determinant role in it [2, 3]. Available antimalarial therapy targets the* Plasmodium*. However, elimination of the parasite does not halt the clinical consequences of disease as surviving patients from severe malaria can develop a range of neurological deficits [4, 5]. In this context, an effective immune response is essential for patient's recovery. Innate response is the major immunity component of patients who have been infected with* Plasmodium* for the first time, being essential to the development of an effective acquired immune response [6].

Recently, a protective role for transient receptor vanilloid 1 (TRPV1), a nonselective cation channel found on both neuronal and nonneuronal cells, was suggested in bacteria-induced sepsis [7–10]. Indeed, in the absence of TRPV1 activation, macrophage functions such as their ability to phagocytose and to release inflammatory mediators (nitric oxide (NO), reactive oxygen species (ROS), and cytokines) are impaired [9]. Also, TRPV1 has been linked to macrophage survival [9]. Evidence suggests a feedback between TRPV1 activation and ROS production may exist; in addition to modulating oxidative stress by downregulating ROS generation, this receptor can be directly activated by hydrogen peroxide (H_2_O_2_) [11] and regulated by superoxide anion (O_2_^−^) release [12–14]. Oxidative stress generation has a direct impact on macrophage-erythrocyte-endothelium interactions and imbalances of this pathway may trigger excessive damage and impaired host's immune response to malaria [15, 16].

Herein, the role of TRPV1 in malaria was investigated for the first time. We used the TRPV1 antagonist, capsazepine, to assess whether TRPV1 is able to modulate the innate immune response to malaria in animals infected with* Plasmodium berghei* ANKA.

## 2. Materials and Methods

### 2.1. Animals

Inbred male C57BL/6 mice (8 weeks old) were used. Mice were obtained from the animal's facility of the Department of Parasitology, Institute of Biomedical Sciences, University of São Paulo. Mice were kept in a climatically controlled environment and given food and water* ad libitum*. All procedures were approved by the Ethics Committee of the University of São Paulo and carried out in accordance with the Brazilian society for animal welfare (SBCAL).

### 2.2. Malaria Induction

Malaria was induced by a single intraperitoneal (i.p.) injection of 10^5^ red blood cells (RBCs) infected with* Plasmodium berghei* ANKA (clone 1.49L) as described by Elias et al. [17]. Parasitaemia was assessed daily in a blood smear stained by Giemsa, by microscopy, from day 5 to day 7 following infection and was expressed as % of infected RBCs. Mice were terminally anaesthetised with a mixture of ketamine (75 mg/kg; Dopalen, Ceva, Brazil) and xylazine (1 mg/kg; i.p.; Sigma-Aldrich, Brazil), and exsanguinated by cardiac puncture on day 7 after infection (premortality end point; [18, 19]). Their blood and spleen were collected for further analysis. The plasma was separated and stored at −70°C for further quantitation of plasma aldehydes and cytokines. Cell population phenotype and activation were evaluated by flow cytometry. Noninfected mice were used as controls.

### 2.3. Pharmacological Treatment

In order to assess the role of TRPV1 in malaria, animals received the TRPV1 antagonist, capsazepine (Sigma-Aldrich, Brazil; *n* = 5, uninfected (control) group and *n* = 8, infected group), intraperitoneally from 24 h following infection, for 6 days (2x day, 50 *μ*g/animal; [9]). Vehicle (10% DMSO in saline, 120 *μ*L/animal; *n* = 5, uninfected (control) group and *n* = 8, infected group) was used as controls.

### 2.4. Flow Cytometry Analysis

Blood and spleen samples from infected and uninfected mice and single-cell suspensions were prepared. Peripheral blood cells were isolated by Percoll gradient (Sigma-Aldrich, Brazil). Spleens were homogenized and passed through a nylon mesh of 70 *μ*m to create a single-cell suspension. Cells were stained with Trypan blue (Sigma-Aldrich, Brazil) and assessed for viability in a haemocytometer. Cells (10^6^) were washed, resuspended in flow cytometry buffer (2% foetal calf serum (Invitrogen, Brazil) in phosphate buffered saline-PBS (Sigma-Aldrich, Brazil)), and stained with directly conjugated monoclonal antibodies (BD Biosciences or eBiosciences, Brazil): anti-F4/80 FITC, anti-IAb PE, anti-Ly6G PerCP, anti-GR1 Pe-Cy7, anti-CD3 PE and APC, anti-NK1.1 FITC, and anti-CD69 PECy5. Events were acquired on a BD FACSCanto (BD Biosciences-Immunocytometry Systems) and analyzed using FlowJo software (Tree Star Inc.). In order to analyze monocyte/macrophage and neutrophil populations, cells expressing CD3^+^, CD4^+^, CD8^+^, and CD19^+^ were gated out and the phenotypic F4/80, GR1, and Ly6G lineage markers were evaluated. Results are expressed as representative two-colour dot-plots as well as number of cells (×10^6^), except for IAb, expressed as mean fluorescence.

### 2.5. Plasma Cytokine Levels

The plasma levels of TNF*α*, IFN*γ*, IL-4, IL-6, IL-2, IL-10, and IL-17 were evaluated by using a cytometric bead array (CBA) mouse Th1/Th2/Th17 cytokine kit (BD Biosciences, Brazil) according to manufacturer's instructions. Analysis was performed on a Facscalibur cytometer flow cytometer (BD Biosciences-Immunocytometry Systems). Results were calculated in CBA FCAP Array software (BD Biosciences, Brazil) as pg/mL and are expressed as fold-increase relative to uninfected controls.

### 2.6. Plasma Aldehydes

Total plasma aldehyde (mainly malondialdehyde) concentrations were quantified as an index of lipid peroxidation and oxidative stress, as previously described [20, 21]. Briefly, 100 *μ*L of sample were incubated with 100 *μ*L of PBS and 400 *μ*L of thiobarbituric acid (0.67%; Sigma-Aldrich, Brazil), at 90°C for 45 min. Samples were then centrifuged at 1,000 xg for 10 min. Three hundred *μ*L of the supernatant was incubated with 300 *μ*L of butanol (Sigma-Aldrich, Brazil) and 30 *μ*L of a saturated solution of sodium chloride (Sigma-Aldrich, Brazil). Samples were mixed in a vortex, centrifuged at 1,000 xg for 2 min and then added to a 96-well plate (200 *μ*L/well). Absorbance was read at 535 and 572 nm and the difference between the absorbance was used to calculate the aldehyde concentrations, using the molar extinction coefficient of the chromophore (1.56 × 10^5^ M^−1^/cm^−1^). Results are expressed as fold-increase relative to uninfected controls.

### 2.7. Data Analysis

The results are presented as the mean ± standard deviation (SD). The percentages of inhibition are reported as mean ± SD of inhibitions obtained in each individual experiment compared with control samples. Statistical comparisons of the data were performed by ANOVA followed by Bonferroni and unpaired *t*-test when appropriate. The *P* values < 0.05 were considered significant.

## 3. Results

### 3.1. Capsazepine Does Not Affect Parasitaemia

**Figure 1:**
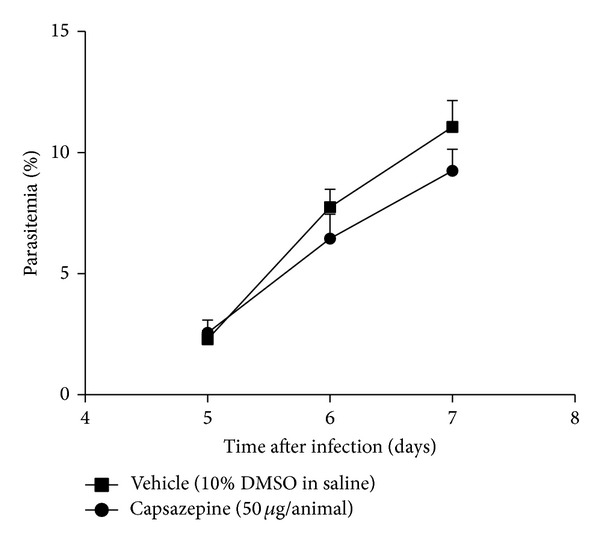
Effect of capsazepine on parasitaemia levels. Parasitaemia was measured daily, from day 5 to day 7 after infection in blood smear samples obtained from C57BL/6 mice infected with* Plasmodium berghei* ANKA (10^5^ infected RBCs/animal; i.p.) treated with either capsazepine (50 *μ*g/animal, 2x day, for 6 days) or vehicle (10% DMSO in saline) from 24 h after infection (*n* = 8 per group).

Figure 1 shows parasitaemia levels up to day 7 after infection, in mice treated with either vehicle or capsazepine. Parasitaemia progressively increased in both groups. Repeated treatment with capsazepine had no effect on parasitaemia. Experiments were performed with a premortality end-point. Thus, no signs of cerebral malaria such as reduced responsiveness to stimulation, ataxia, respiratory distress or prostration, paralysis, and convulsions were observed in either capsazepine- or vehicle-infected mice. However, both groups of animals displayed piloerection and/or abnormal posture, as a result of infection.

### 3.2. Capsazepine Alters Circulating Monocyte but Not Spleen Macrophage Population Number and Activation

**Figure 2:**
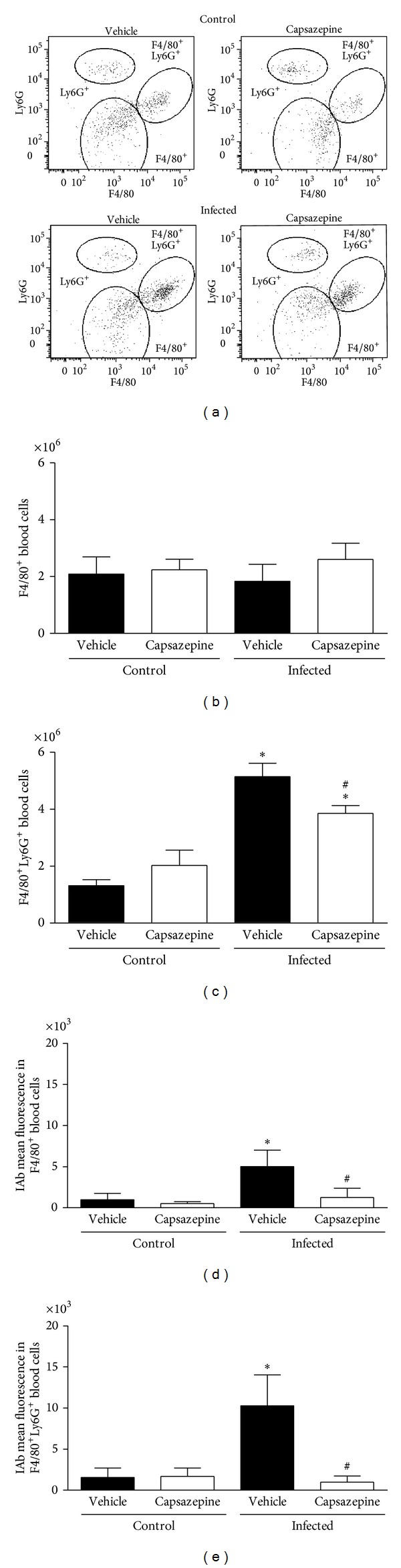
Effect of capsazepine on peripheral blood F4/80^+^, F4/80^+^Ly6G^+^, and Ly6G^+^ cells. (a) Representative two-colour dot-plots for peripheral blood F4/80^+^, F4/80^+^Ly6G^+^, and Ly6G^+^ cell populations from uninfected and* Plasmodium berghei* ANKA-infected mice (10^5^ infected RBCs/animal; i.p.). Circulating (b) F4/80^+^ and (c) F4/80^+^Ly6G^+^ cell numbers in uninfected and* Plasmodium berghei* ANKA-infected mice (10^5^ infected RBCs/animal; i.p.). Expression of IAb (mean fluorescence) on circulating (d) F4/80^+^ and (e) F4/80^+^Ly6G^+^ cell populations. Capsazepine (50 *μ*g/animal, 2x day, for 6 days) was administered from 24 h infection. Vehicle- (10% DMSO in saline) treated animals were used as controls. Data are expressed as mean ± SD, *n* = 5 per group.  *P < 0.05 compared with respective uninfected (control) groups;  ^#^ *P* < 0.05 compared with vehicle-treated infected group.

As represented in Figures 2(a)–2(e), three distinct populations of peripheral blood leukocytes were detected in all groups of uninfected and infected animals: F4/80^+^, F4/80^+^Ly6G^+^ and Ly6G^+^ cells. Malaria induction had no effect on Ly6G^+^ cell population as no statistical significance was found between any of the evaluated groups. Mean ± SD values for Ly6G^+^ populations are as follows: vehicle-uninfected group 2.0 ± 0.6, capsazepine uninfected group 1.3 ± 0.4, vehicle-infected group 0.9 ± 1.1, and capsazepine infected group 1.3 ± 0.8. On the other hand,* P. berghei* ANKA infection increased F4/80^+^Ly6G^+^ cell numbers in both vehicle- (3.9-fold increase) and capsazepine- (1.9-fold increase) treated groups when compared to uninfected controls, whilst no effects were observed on F4/80^+^ cell population (Figures 2(b) and 2(c)). In addition, malaria caused increased activation of both F4/80^+^ (5.1-fold increase) and F4/80^+^Ly6G^+^ (6.6-fold increase) circulating cells when compared to uninfected animals, as denoted by expression of IAb on these cells (Figures 2(d) and 2(e)). Repeated administration of capsazepine in infected animals caused reduction of F4/80^+^Ly6G^+^ population expansion by 25.0 ± 5.2% but did not affect F4/80^+^ cell numbers (Figures 2(b) and 2(c)). In addition,* P. berghei *ANKA-induced activation of F4/80^+^ and F4/80^+^Ly6G^+^ cells was halted by capsazepine. As depicted in Figures 2(d) and 2(e), capsazepine treatment reduced by 75 ± 22.7% and 90.3 ± 7.1%, the expression of IAb on F4/80^+^ and F4/80^+^Ly6G^+^ cells, respectively, when compared to vehicle-treated infected controls. As infection raised the number of circulating F4/80^+^Ly6G^+^ cells, a decline was noticed in the GR1^+^ cell population (34.1 ± 15.1%). However, infected mice treated with capsazepine exhibited a higher number of these cells when compared with both their uninfected- (1.8-fold increase) and infected- (2.4-fold increase) control animals. Mean ± SD values for GR1^+^ populations are as follows: vehicle-uninfected group 2.9 ± 0.6, capsazepine uninfected group 2.5 ± 0.4, vehicle-infected group 1.9 ± 0.4, and capsazepine infected group 4.6 ± 0.5  (*P* < 0.05). Capsazepine treatment in uninfected animals had no effects in regard to expansion or activation of both F4/80^+^ and F4/80^+^Ly6G^+^ cells (Figures 2(a)–2(e)) and neither on GR1^+^ cells.

**Figure 3:**
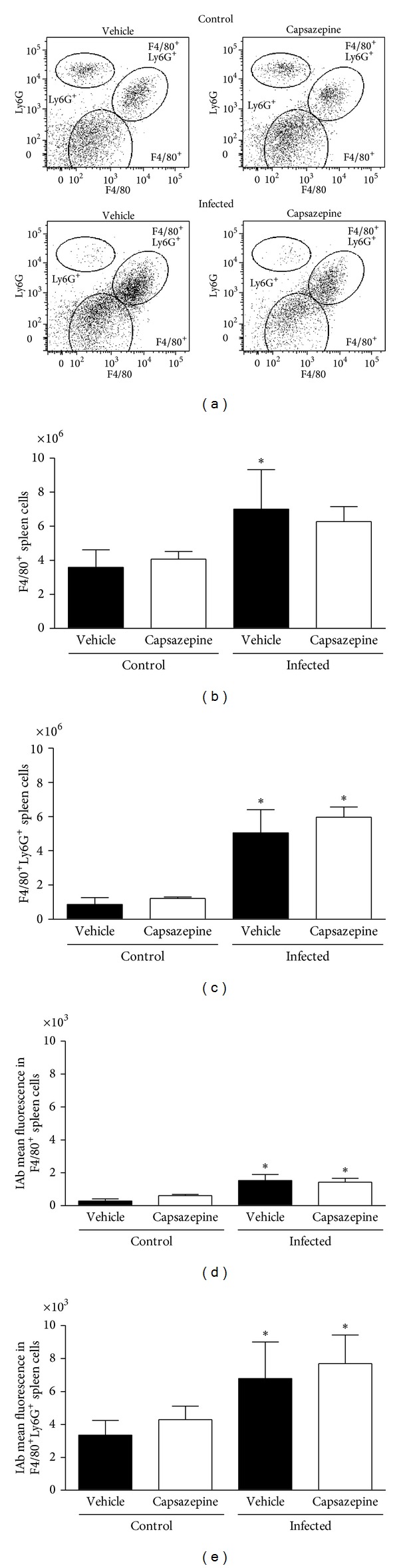
Effect of capsazepine on spleen F4/80^+^, F4/80^+^Ly6G^+^, and Ly6G^+^ cells. (a) Representative two-colour dot-plots for spleen F4/80^+^, F4/80^+^Ly6G^+^, and Ly6G^+^ cell populations from uninfected and* Plasmodium berghei* ANKA-infected mice (10^5^ infected RBCs/animal; i.p.). Spleen (b) F4/80^+^ and (c) F4/80^+^Ly6G^+^ cell numbers in uninfected and* Plasmodium berghei* ANKA-infected mice (10^5^ infected RBCs/animal; i.p.). Expression of IAb (mean fluorescence) on spleen (d) F4/80^+^ and (e) F4/80^+^Ly6G^+^ cell populations. Capsazepine (50 *μ*g/animal, 2x day, for 6 days) was administered from 24 h after infection. Vehicle- (10% DMSO in saline) treated animals were used as controls. Data are expressed as mean ± SD, *n* = 5 per group.  *P < 0.05 compared with respective uninfected (control) groups.

Similarly, F4/80^+^, F4/80^+^Ly6G^+^ and Ly6G^+^ cells were detected in spleen samples obtained from both infected and uninfected mice whether or not they were treated with capsazepine (Figures 3(a)–3(e)). As observed for circulating Ly6G^+^ cells, malaria induction had no effects on spleen Ly6G^+^ cells. Mean ± SD values for spleen Ly6G^+^ populations are as follows: vehicle-uninfected group 0.7 ± 0.2, capsazepine uninfected group 1.1 ± 0.4, vehicle-infected group 1.0 ± 0.6, and capsazepine infected group 0.9 ± 0.3. However,* P. berghei* ANKA injection raised the numbers of F4/80^+^ and F4/80^+^Ly6G^+^ cells in both vehicle- (2.0- and 5.8-fold increase, resp.) and capsazepine- (1.5- and 4.9-fold increase, resp.) treated groups when compared to their respective uninfected controls (Figures 3(b) and 3(c)). Also,* P. berghei* ANKA infection augmented F4/80^+^ and F4/80^+^Ly6G^+^ cells in both vehicle- (5.5- and 2.0-fold increase, resp.) and capsazepine- (2.3- and 1.8-fold increase, resp.) treated groups when compared to their respective uninfected controls (Figures 3(d) and 3(e)). Capsazepine had no effects on the number or activation of spleen F4/80^+^ and F4/80^+^Ly6G^+^ cells (Figures 3(a)–3(e)). Spleen GR1^+^ cell population remained unaltered irrespective of treatments and mean ± SD values are as follows: vehicle-uninfected group 0.9 ± 0.2, capsazepine uninfected group 1.3 ± 0.6, vehicle-infected group 0.8 ± 0.3, and capsazepine infected group 1.3 ± 0.4. Moreover, capsazepine had no effects on F4/80^+^ and F4/80^+^Ly6G^+^ cells (Figures 3(a)–3(e)) when administered to uninfected mice.

### 3.3. Capsazepine Modulates Blood and Spleen NK and NKT Population Number and Activation

**Figure 4:**
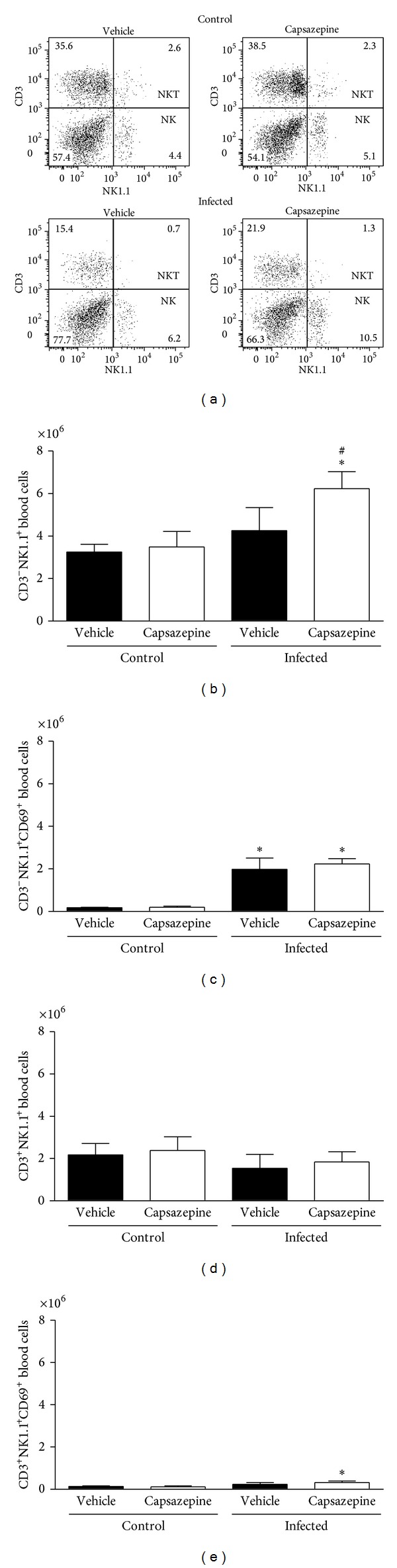
Effect of capsazepine on peripheral blood CD3^−^NK1.1^+^ and CD3^+^NK1.1^+^ cells. (a) Representative two-colour dot-plots for peripheral blood CD3^−^NK1.1^+^ (NK) and CD3^+^NK1.1^+^ (NKT) cell populations from uninfected and* Plasmodium berghei* ANKA-infected mice (10^5^ infected RBCs/animal; i.p.). Circulating (b) CD3^−^NK1.1^+^ and (c) CD3^−^NK1.1^+^cell numbers in uninfected and* Plasmodium berghei* ANKA-infected mice (10^5^ infected RBCs/animal; i.p.). Expression of CD69 on circulating (d) CD3^−^NK1.1^+^ and (e) CD3^−^NK1.1^+^cells. Capsazepine (50 *μ*g/animal, 2x day, for 6 days) was administered from 24 h after infection. Vehicle- (10% DMSO in saline) treated animals were used as controls. Data are expressed as mean ± SD, *n* = 5 per group.  *P < 0.05 compared with respective uninfected (control) groups.

We also evaluated the effects of capsazepine on circulating and spleen NK (CD3^−^NK1.1^+^) and NKT (CD3^+^NK1.1^+^) cells. Peripheral blood NK and NKT cells were detected in all groups of uninfected and infected animals (Figures 4(a)–4(e)). Malaria induction had no effects on either NK or NKT cell numbers when compared to uninfected control mice (Figures 4(b) and 4(d)). On the other hand, NK, but not NKT activation via CD69 expression, was increased in both vehicle- (11.1-fold increase) and capsazepine- (11.5-fold increase) treated groups when compared to their respective uninfected controls (Figure 4(c)). However, capsazepine treatment significantly increased NK cell population (Figure 4(b)) when compared to either its uninfected control group (1.8-fold increase) or vehicle-treated infected animals (1.5-fold increase). Also, capsazepine was able to enhance activation of NKT-infected cells (2.7-fold increase; Figure 4(e)). Importantly, NK cells account for the majority of circulating and activated NK1.1^+^ cells in infected animals (Figure 4).

**Figure 5:**
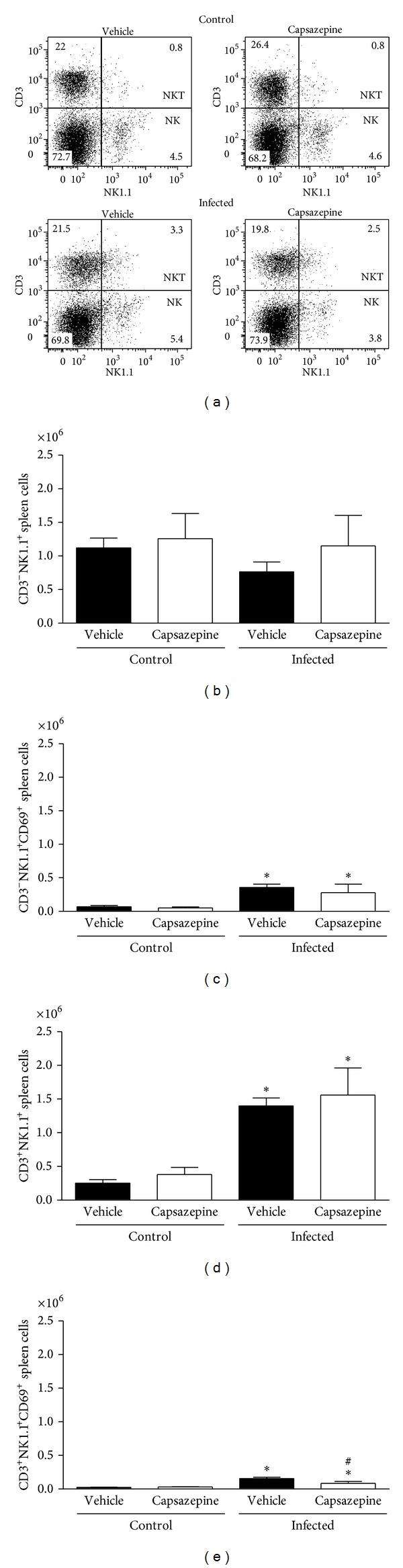
Effect of capsazepine on spleen CD3^−^NK1.1^+^ and CD3^+^NK1.1^+^ cells. (a) Representative two-colour dot-plots for spleen CD3^−^NK1.1^+^ (NK) and CD3^+^NK1.1^+^ (NKT) cell populations from uninfected and* Plasmodium berghei* ANKA-infected mice (10^5^ infected RBCs/animal; i.p.). Spleen (b) CD3^−^NK1.1^+^ and (c) CD3^−^NK1.1^+^ cell numbers in uninfected and* Plasmodium berghei* ANKA-infected mice (10^5^ infected RBCs/animal; i.p.). Expression of CD69 on circulating (d) CD3^−^NK1.1^+^ and (e) CD3^−^NK1.1^+^ cells. Capsazepine (50 *μ*g/animal, 2x day, for 6 days) was administered from 24 h after infection. Vehicle- (10% DMSO in saline) treated animals were used as controls. Data are expressed as mean ± SD, *n* = 5 per group.  *P < 0.05 compared with respective uninfected (control) groups.

Similarly, spleen NK and NKT cells were detected in all groups of uninfected and infected animals (Figures 5(a)–5(e)). Malaria induction caused spleen NKT but not NK cell population expansion when compared to uninfected control mice (Figures 5(b) and 5(d)). This was observed for both vehicle- (5.6-fold increase) and capsazepine- (4.1-fold increase) treated mice (Figure 5(d)). Figures 5(c) and 5(e) demonstrate that CD69 expression was augmented on both spleen NK and NKT cells obtained from infected mice treated with either vehicle (5.3- and 6.9-fold increase, resp.) or capsazepine (5.6- and 3.1-fold increase, resp.). However, NKT activation was 45.7 ± 17.5% lower in capsazepine-treated mice when compared to vehicle-infected controls (Figure 5(e)). Capsazepine treatment in infected mice had no effects on spleen NK and NKT cell numbers or NK activation (Figure 5). Similarly, capsazepine did not alter spleen NK and NKT profile (cell number and activation) in samples obtained from uninfected mice (Figure 5).

### 3.4. Capsazepine Reduces Lipid Peroxidation and Plasma TNF*α* Levels in* P. berghei* ANKA-Infected Mice

**Figure 6:**
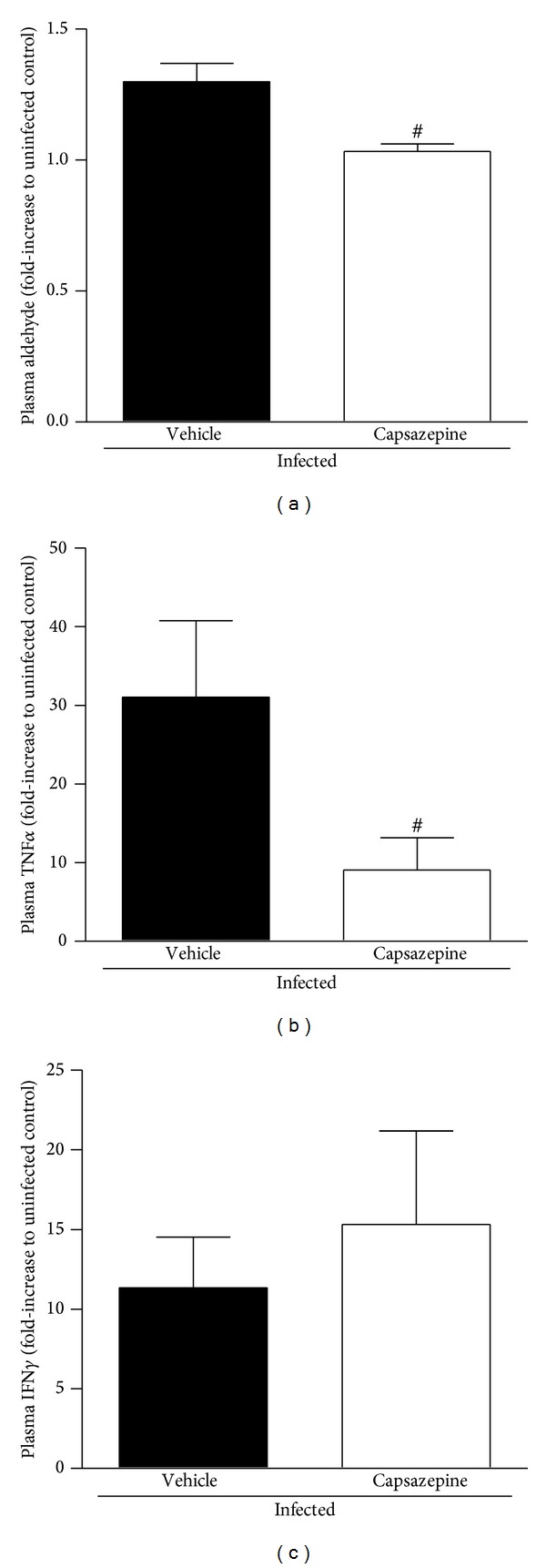
Effect of capsazepine on systemic lipid peroxidation and cytokine release. (a) Aldehyde; (b) TNF*α* and (c) IFN*γ* levels in plasma samples obtained from uninfected and* Plasmodium berghei* ANKA-infected mice (10^5^ infected RBCs/animal; i.p.). Capsazepine (50 *μ*g/animal, 2x day, for 6 days) was administered from 24 h after infection. Vehicle- (10% DMSO in saline) treated animals were used as controls. Data are expressed as mean ± SD, *n* = 5–8 per group.  ^#^ *P* < 0.05 compared with vehicle-treated infected group.

*P. berghei* ANKA infection increased lipid peroxidation in both vehicle- and capsazepine-treated mice, as demonstrated by the levels of plasma aldehydes (Figure 6(a)). However, this increase was less pronounced in the capsazepine-treated group (20.5 ± 6.4% reduction). Malaria also triggered the release of TNF*α* and IFN*γ* in both infected groups (Figures 6(b) and 6(c)). TNF*α* production was markedly reduced by capsazepine treatment (70.8 ± 14.5%; Figure 6(b)). On the other hand, capsazepine treatment did not affect IFN*γ* release triggered by malaria (Figure 6(c)). Vehicle- and capsazepine-treated uninfected mice exhibited similar levels of plasma aldehydes, TNF*α*, and IFN*γ* (data not shown). Production of IL-4, IL-6, IL-2, IL-10, and IL-17 was not detected in any of the evaluated groups.

## 4. Discussion

Since its discovery, evidence has accumulated that TRPV1 has the potential to play a key role in a variety of pathologies, especially those associated with imbalances of the immune and inflammatory response, such as asthma [22–24] and rheumatoid arthritis [25–27]. Recent reports demonstrated that TRPV1 is expressed on immune cells such as macrophages and peripheral-blood mononuclear cells in inflammatory conditions [9, 28–30]. More recently, TRPV1 was suggested to modulate a range of macrophage-mediated responses to bacterial infection [9]. Indeed, TRPV1 deletion or antagonism has been associated with poorer outcome of experimental sepsis as TRPV1 blockade increases pathogen load and also facilitates the transition from a local to a systemic inflammatory response to bacteria [7, 9, 10, 31–34]. In addition, TRPV1 knockout (TRPV1 KO) mice challenged with intestinal bacteria or LPS present with a dysregulated production of inflammatory mediators, including NO, ROS, and cytokines such as TNF*α*, IL-10, and IL-6 [7–9]. So far, there are no reports of TRPV1 playing any roles in the immune response to other pathogens.

Here, we show for the first time that TRPV1 antagonism by capsazepine, a nonselective antagonist, modulates the innate immune response to malaria. Reports have shown that capsazepine presents species- and modality-specific activity on TRPV1 and also inhibits acetylcholine receptors, voltage-gated calcium channels, and hyperpolarization-activated cyclic nucleotide-gated channels, in addition to TRPV1 [35–38]. However, it is important to highlight that capsazepine was administered in this study, as described by Fernandes and collaborators [9] who showed that repeated treatment with this drug* in vivo* produces a similar profile to that of TRPV1 KO mice in response to bacterial infection [9]. Herein, we induced malaria by injecting* P. berghei* ANKA in a susceptible strain of mice known to develop cerebral malaria-like symptoms and to present with 60–100% mortality within the second week following injection (for review see [19]). Our studies were carried out at a premortality end-point, that is, 7 days following infection. At this time point, parasitaemia had reached 11%, in agreement with previous studies [17, 22].

Evidence suggests that the innate immune response plays an important role during the early phase of infection, with activated monocytes and neutrophils releasing nonspecific inflammatory mediators such as ROS and cytokines [2, 39] and exerting their roles as phagocytes and antigen-presenting cells when in contact with circulating infected RBCs [40]. Indeed, phagocytosis of infected RBCs by peripheral blood and tissue phagocytes is suggested to be the major mechanism of* Plasmodium* removal [41]. As a result of this interaction, phagocytes may damage the endothelium, thus contributing to the collapse of the circulation and in the case of cerebral malaria, damaging the brain microvasculature [4, 42]. During infection, monocytes differentiate into macrophages in the spleen and also in the brain, becoming available in the brain microvasculature [4]. Activated macrophages contribute to pathogen clearance and activation of lymphocytes, in an attempt to stimulate the generation of an acquired immune response capable of improving parasite removal and fighting a secondary infection [2, 43].

As previously mentioned, TRPV1 can be found on immune cells. It is then expected that antagonism of TRPV1 channels expressed on these cells would affect their phenotypes. Our results show that at 7 days after infection, there is activation of both monocytes and spleen macrophages (F4/80^+^). Interestingly, we detected a group of circulating F4/80^+^Ly6G^+^ cells which became markedly expanded and activated (as denoted by IAb expression) following infection. This is the first report to our knowledge of their contribution to malaria. However, it was recently suggested in a model of infection caused by vaccinia virus inoculation in C57BL/6 mice that F4/80^+^Ly6G^+^ cells are indeed monocytes with a great capacity of producing ROS and IFN*γ* whilst Ly6G^−^ monocytes produce NO and TNF*α* [44]. In addition, the same study suggested that F4/80^+^Ly6G^+^ cells replace Ly6G^−^ monocytes as infection progresses. Herein, we show the existence of F4/80^+^Ly6G^+^ cells also in the spleen and that they exhibit a similar profile (in terms of number and activation pattern) to that of Ly6G^−^ cells in this tissue. As the population of F4/80^+^Ly6G^+^ cells rises, GR1^+^ cells (myeloid-derived suppressor cells) decline with infection. This is expected as an increase of mature monocytes and neutrophils normally occurs as a result of infection [45, 46]. However, at 7 days after infection, these changes in the balance and/or activation of F4/80^+^, F4/80^+^Ly6G^+^, and GR1^+^ cells could not be noticed at spleen level. However, it is possible that these alterations may occur in this organ at a different time point not addressed by this study.

Repeated treatment with capsazepine caused inhibition of activation of both F4/80^+^ and F4/80^+^Ly6G^+^ cells as well as reduction of the number of circulating F4/80^+^Ly6G^+^ cells in infected mice. Interestingly, GR1^+^ cell number is markedly increased in the same group of animals, suggesting that, as capsazepine shuts down F4/80^+^- and F4/80^+^Ly6G^+^-mediated responses, either by deactivating them or decreasing their availability in the circulation, these cells are progressively replaced by GR1^+^ cells in order to restore the immune responses that may be dependent on F4/80^+^- and F4/80^+^Ly6G^+^ monocytes. Indeed, GR1^+^ cells are suggested to be recruited in order to compensate “the loss” of monocytes that undergo polarization following* Plasmodium* or bacterial infection [47, 48]. These cells are also called regulatory monocytes/macrophages and have a potent ability to suppress T cell proliferation and Th1 responses [49]. On the other hand, the definite role of these cells in critical illness is still of debate (for review see [47]).

To evaluate the impact this shift on monocyte profile had on inflammatory mediator release, we measured the levels of plasma aldehydes (index of lipid peroxidation secondary to oxidative stress) and cytokines such as TNF*α* and IFN*γ*. Indeed, high systemic levels of inflammatory mediators such as TNF*α*, IFN*γ*, and aldehydes are correlated with severe malaria in humans [50]. In fact, lipid peroxidation is produced during malaria as a result of the interactions between monocytes/macrophages and infected erythrocytes, and also the endothelium [51, 52]. In addition, cytokines are also released in response to malaria, triggering suppression of erythropoiesis and activation of a variety of circulating and spleen cells [2, 53, 54]. We found that whilst diminished levels of lipid peroxidation-derived aldehydes and TNF*α* were detected in animals with malaria that had been treated with capsazepine, IFN*γ* production remained similar to that observed for samples obtained from vehicle-infected mice. A reduction of oxidative stress was expected as capsazepine was previously suggested to inhibit oxidative stress in cultured RAW264 monocytes/macrophages in a TRPV1 independent manner, although this data was obtained from cells that had not been challenged with any pathogen product [55]. Later, TRPV1 deletion was shown to decrease ROS production by macrophages in sepsis [9] and to modulate the release of these mediators in other inflammatory conditions [14, 56]. However, loss of TRPV1 function has been associated with increased production of TNF*α* upon bacterial infection [7, 9, 32]. It is possible that TRPV1 differently modulates monocytes and macrophages with respect to their ability to produce TNF*α*. Also, TRPV1 effects on TNF*α* production may vary at different stages of malaria.

NK and NKT cells are important effectors of the innate immune response to malaria, directly recognizing* Plasmodium*-infected RBCs and malarial antigens, in addition to producing IFN*γ* in order to contain parasitaemia [2, 18, 40]. Indeed, these cells rise early during malaria and have been suggested to mediate the differentiation of Th1/Th2 responses and to be essential for the trafficking of leukocytes to the brain in cerebral malaria (for review see [2]). In addition, similarly to macrophages, they can accumulate into the brain during cerebral malaria, contributing to a worse outcome [18]. NK cells have also been linked to dendritic cell maturation and T cell activation in the spleen by releasing of cytokines [53, 57]. Also, evidence has shown that during malaria, NK cells may undergo an intense turnover or even migrate out of the spleen [18]. This was investigated at a similar time-point to that used in our study (1 week following infection). Here, we assessed the dynamics between circulating and spleen NK and NKT cells. We found that* P. berghei *ANKA-induced infection caused expansion of spleen NKT but not NK cell population. This was not accompanied by any change on circulating NK and NKT cell numbers. It is possible that at this time-point, NK and NKT cells have already migrated to the brain. Indeed, Hansen and collaborators showed that NK cells accumulate into the brain of C57BL/6 mice as early as 4 days following infection with* P. berghei* ANKA, triggering the migration of T cells to the brain microvasculature [18]. On the other hand, we show that both NK and NKT cells became activated in response to infection, with NK cells representing the majority of activated cells. Disappointingly, expansion of spleen NKT cells did not translate in their activation as only few of these cells expressed CD69. Capsazepine treatment in infected mice led to a further expansion of the circulating NK population, but its activation was similar to that of vehicle-infected group. In addition, capsazepine treatment reduced by half the activation of spleen NKT cells when compared to its infected-control. We would expect that the reduction of activation of spleen NKT cells and F4/80^+^Ly6G^+^ monocytes by capsazepine would impair IFN*γ* release. However, we show capsazepine inhibitory effects on these populations do not affect IFN*γ* production in malaria, indicating that either TRPV1 may not play a role on IFN*γ* production or NK cells become the sole source of IFN*γ* once TRPV1 is blocked. In addition, the lack of effect of capsazepine on parasitaemia may be related to the similar levels of IFN*γ* detected in both groups of animals.

Our study provides the first evidence that TRPV1 modulates malaria by mediating innate immune response, specifically by interfering with the expansion and activation of effector cells, especially monocytes. We also show that TRPV1 regulates the immunological balance between different monocyte populations in addition to modulating mediator release by them. It is possible that blocking TRPV1 may be either beneficial, as a reduction of oxidative stress may reflect on reduced vascular dysfunction, or deleterious, as impairment of innate response may lead to an inefficient removal of the parasite in addition to an inefficient acquired immune response to malaria. However, the impact TRPV1 antagonism may have on severe malaria outcome is of importance and remains to be investigated.
